# Imaging of Myocardial Oxidative Metabolism in Heart Failure

**DOI:** 10.1007/s12410-013-9244-y

**Published:** 2013-12-27

**Authors:** Masanao Naya, Nagara Tamaki

**Affiliations:** 1 Department of Cardiology, Hokkaido University School of Medicine, Sapporo, 060 Japan; 2 Department of Nuclear MedicineHokkaido, University School of Medicine, Kita-15, Nishi-7, Kita-ku, 060 Japan

**Keywords:** Oxidative metabolism, Heart failure, C-11 acetate, Positron emission tomography, Metabolic work index

## Abstract

Metabolic imaging has a potential for better understanding of pathophysiology of heart failure. C-11 acetate is taken up by the heart, rapidly converted to acetylCoA and readily metabolized to C-11 CO_2_ through TCA cycle with oxidative phosphorylation. Thus, the myocardial turnover rate of this tracer is tightly correlated with its clearance of C-11 CO_2_, reflecting overall oxidative metabolism. The heart relies on aerobic oxidative substrate for the generation of ATP, which is required to maintain its contractile function. The progression to heart failure is associated with a gradual decline in the activity of mitochondrial respiratory pathways, leading to diminished capacity for ATP production. The work metabolic index can also be estimated by the combination of C-11 acetate PET and hemodynamics by echocardiography, the metabolic index is a significant marker to understand the pathophysiology of heart failure as well as myocardial oxidative metabolism.

## Introduction

Metabolic imaging has a potential for better understanding of pathophysiology of heart failure. The advantage of tracer techniques is to use a radiolabeled compound for in vivo quantification of specific biological processes. With introduction of positron emission tomography (PET), the spectrum of in vivo tissue characterization has been widened with use of physiological tracers labeled with C-11, N-13, O-15, and F-18 which allow the synthesis of naturally occurring and biologically active compounds. The importance of energy metabolism in maintaining the integrity of cardiac performance has been increasingly recognized with PET.

C-11 palmitate and F-18 fluorodeoxyglucose (FDG) are used to estimate myocardial metabolism with myocardial energy substrate such as fatty acid and glucose utilizations, respectively by PET. On the other hand, a direct estimate of tricarboxylic acid (TCA) cycle is an alternative approach for assessing myocardial metabolism. C-11 acetate has been proposed as a PET tracer to probe myocardial oxidative metabolism with PET. This tracer, a simple two-carbon carboxylic acid, can be rapidly prepared in high yields by the simple one step C-11 carboxylation of the appropriate Grinard reagent, methylmagnesium bromide [1].

C-11 acetate is taken up by the heart, rapidly converted to acetylCoA and readily metabolized to CO2 through TCA cycle with oxidative phosphorylation. Thus, the myocardial turnover rate of this tracer is tightly correlated with its clearance of C-11 CO2, reflecting overall oxidative metabolism. The myocardial clearance rate following intravenous administration of C-11 acetate correlates closely with myocardial oxygen consumption measured by arterial-venous difference of oxygen in isolated heart preparation during ischemia and reperfusion [2, 3]. While metabolic studies with C-11 palmitate or FDG studies are quite dependent on plasma substrate levels, C-11 acetate metabolism is independent of concentration of energy substrates for the myocardium [4, 5].

## How to Analyze Oxidative Metabolism

**Fig. 1:**
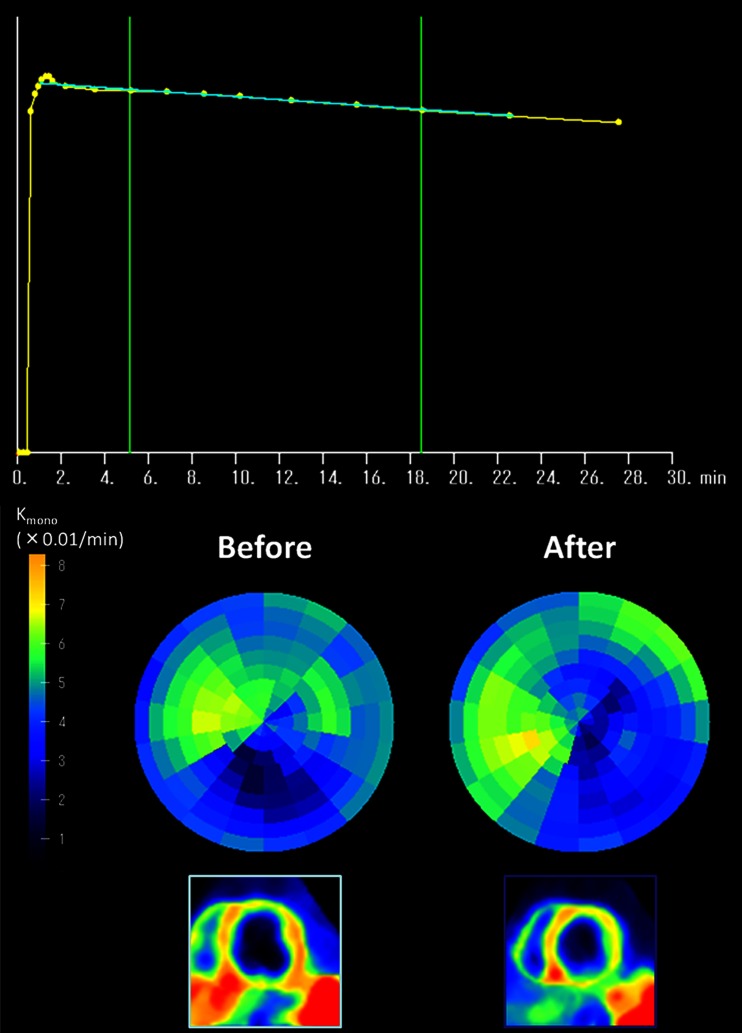
Monoexponential clearance rate of C-11 acetate for the myocardium (Kmono) (a). Polar map in patients with dilated cardiomyopathy. Kmono before and after surgery (b) and C-11 acetate imaging (early phase) of short-axis view in the midlevel before and after surgery (c). Kmono value did not change, whereas left ventricular size decreased after surgery [43••]

Following administration of 10–20 mCi (370-740 MBq) of C-11 acetate, dynamic PET acquisition is performed over 20–30 minutes. A rapid clearance of blood pool activity and high myocardial uptake create high quality myocardial images with high target-to-background ratio a few minutes after tracer administration. A uniform washout from the left ventricular myocardium is obtained in the normal subjects, indicating a homogeneous oxidative metabolism. A mono-exponential or bi-exponential least square fitting can be applied to the regional myocardial time activity curves. The Kmono or K1 values are used as indices of regional myocardial oxidative metabolism. This is a simple method for the quantitative analysis of washout kinetics of this tracer (Fig. 1) [5–7].

In vivo canine studies with C-11 acetate and PET delineated the chronology of restoration of oxidative metabolism with respect to recovery of systolic function and changes in substrate utilization after reperfusion [7–9]. Following a short period of ischemia, both oxidative metabolism and systolic function were reduced but both parameters increased in response to inotropic stimulation by pacing, indicating stunned myocardium showing both oxidative and functional reserve [10]. Following longer periods (1–3 hrs) of ischemia, oxidative metabolism did not return to baseline levels until at least 2–6 weeks which is paralleled with the results of recovery of systolic function [11]. These experimental results indicate the importance of the recovery of oxidative metabolism in predicting the recovery of systolic function following myocardial ischemia.

The human experiments also showed the close estimates of myocardial oxygen consumption by the C-11 acetate kinetic study [9]. One of the major importances of this analysis is that the relationship of the turnover rate of this tracer is relatively insensitive to changes in the substrate environment.

When the estimate of myocardial oxygen consumption by C-11 acetate PET is compared with measurement of left ventricular external work, work metabolic index can be calculated [11–13]. Mechanical efficiency is the external work corrected for total myocardial oxygen consumption. Noninvasively, efficiency can be estimated by combining echocardiographic stroke work, mean blood pressure, and oxygen consumption estimated by C-11 acetate PET [11–13]. This approach indicates a significant promise as a means to delineate mechanics responsible for cardiac function and treatment effect in patients with poor ventricular dysfunction.

## Clinical Applications for Ischemic Heart Disease

C-11 acetate PET permits observation of the effects of acute ischemia on oxidative metabolism in a clinical setting in patients with acute myocardial infarction similar to the experimental model [14]. In addition, the time course of restoration of metabolism can be analyzed in relation to recovery of myocardial perfusion and function after thrombolysis. Henes et al. [15] indicated markedly reduced metabolism in the central zone of infarction with no change over time in patients with conservative treatment. In contrast, in patients with acute myocardial infarction with successful thrombolysis, perfusion was normalized within 24 hours, whereas oxidative metabolism was impaired initially and increased slowly over time. In addition, the improvement in regional systolic function was only seen in those with normal perfusion and improvement in metabolism. Kalff et al. [16] indicated the reduction of oxidative metabolism in normal myocardium by beta-blockade, indicating myocardial protection. Czernin et al. [17] demonstrated the preserved oxidative metabolism in the areas with flow and glucose metabolism mismatch in patients with recent myocardial infarction, suggesting regional increase in oxygen extraction in combination of enhanced glucose metabolism. These data indicate frequent dissociation of regional perfusion and oxidative metabolism after myocardial infarction, and potential value for assessing oxidative metabolism in comparison with perfusion for precise assessment of tissue function in infarcted and peri-infarcted areas. Vanoverschelde et al. [18] showed that regional flow and oxidative metabolism were well preserved in the chronically dysfunctional areas without prior myocardial infarction. They proposed that such areas result from repeated episodes of ischemia.

Assessment of myocardial viability has been focused in the clinical studies using PET. Because of the importance of preservation of oxidative metabolism for the recovery of ventricular dysfunction after ischemia, C-11 acetate PET has been applied for tissue viability analysis. Gropler et al. first reported value of oxidative metabolism assessed by dynamic PET with C-11 acetate for differentiation of viable from non-viable myocardium [19]. The preservation of oxidative metabolism may be required for recovery of function after coronary revascularization in patients with chronic coronary artery disease [20, 21]. This concept has been extended to patients after acute myocardial infarction. Hicks et al. [22] explored the value of C-11 acetate for predicting long-term outcome in regional contractile function in infarct territories. This study employs the initial uptake of this tracer as a measure of relative regional blood flow [23, 24] to compare relative acetate clearance. Concordant reduction of blood flow and oxidative metabolism may indicate nonviable myocardium, and thus, lack of long-term improvement in contractile function. On the contrary, those with greater reduction of oxidative metabolism than blood flow appeared to predict future improvement in contractile function. Wolpers et al. [25] indicated a certain threshold of regional perfusion and oxidative metabolism for recovery of regional dysfunction. The areas beyond the threshold may be irreversibly injured myocardium. Hicks et al. [22], on the other hand, showed significant overlap of absolute values of oxidative metabolism between reversible and nonreversible segments after acute myocardial infarction. Comparison of regional perfusion and oxidative metabolism was more predictive of recovery in contractive function.

In patients with left ventricular dysfunction in patients with coronary artery disease, assessment of oxidative metabolism provided an incremental increase in accuracy compared with glucose metabolic study [26, 27]. This superiority of oxidative metabolism over glucose metabolism may be derived from the inability of FDG kinetics to separate the oxidative from nonoxidative component of glucose metabolism. In addition, assessment of oxidative metabolism requires no standardization of energy substrates. On the other hand, Vanoverschelde et al. [28] in the study of patients with reperfused myocardial infarction showed the reduction of oxidative metabolism in proportion of residual myocardial blood flow and no difference in the metabolic value with and without flow-FDG mismatch. According to their findings, oxidative metabolism may not provide additional independent information regarding myocardial viability over the combined evaluation of residual flow and glucose metabolism. However, many more case studies are required to confirm these findings.

PET study with C-11 acetate at rest and dobutamine infusion provides promising approach for assessment of oxidative metabolic reserve [5, 29]. Hata et al. [30] reported that oxidative metabolic reserve after low-dose dobutamine infusion was the better marker of recovery of regional function than the resting oxidative metabolism. On the other hand, Yoshinaga et al. [31] demonstrated reduced oxidative metabolic reserve despite preserved glucose utilization in severely dysfunctional myocardium. Such decrease in oxidative metabolic reserve may come from impaired mitochondrial response to inotropic stimulation. Further analysis is warranted to investigate mitochondrial function in relation to inotropic reserve as well as functional analysis before and after intervention may play an important role for assessing tissue viability.

## Clinical Applications for Heart Failure

The heart failure has a significant impact on quality of life and prognosis. The heart relies almost exclusively on aerobic oxidative substrate for the generation of ATP, which is required to maintain its contractile function. The progression to heart failure is associated with a gradual decline in the activity of mitochondrial respiratory pathways, leading to diminished capacity for ATP production [32]. For the better understanding of the pathophysiology, it is important to assess myocardial oxidative metabolism.

Abnormal oxidative metabolism is often observed in patients with cardiomyopathy. Tadamura et al. [33] showed decreased Kmono values both in the hypertrophic and non-hypertrophic myocardium, indicating regional decrease in oxidative metabolism in patients with hypertrophic cardiomyopathy. Some of these areas were associated with enhanced FDG uptake, whereas others showed concordant decrease in FDG uptake. These findings were not related with cardiac symptoms or familial history of cardiomyopathy. In the study of patients with dilated cardiomyopathy, Bach et al. [34] indicated heterogeneity of regional oxidative metabolism in proportion to heterogeneous ventricular dysfunction on echocardiography. Heterogeneous reduction of oxidative metabolism was also noted not only in ischemic cardiomyopathy but also in dilated cardiomyopathy [35]. If particular, severe reduction of oxidative metabolism was noted in septal regions as compared to other regions in patients with left bundle branch block [35].

### Aortic Stenosis

**Fig. 2:**
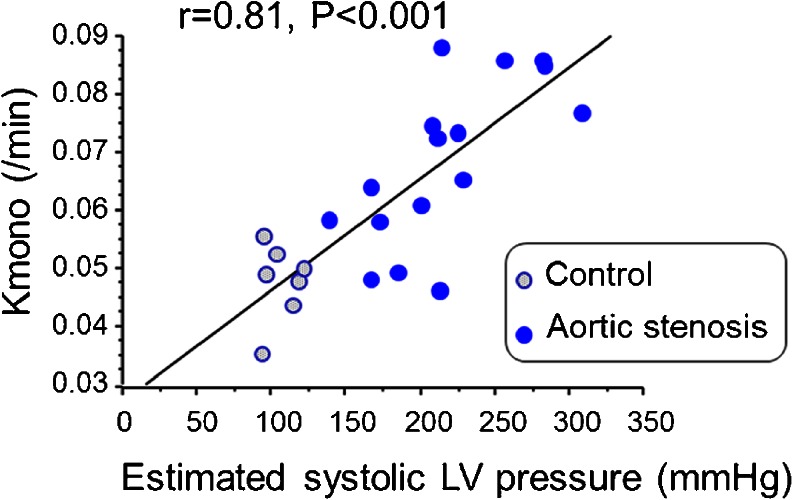
Relationship between Kmono and systolic LV pressure estimated by echocardiography and systolic blood pressure in healthy controls (n = 7) and patients with AS (n = 16) [37••]

**Fig. 3:**
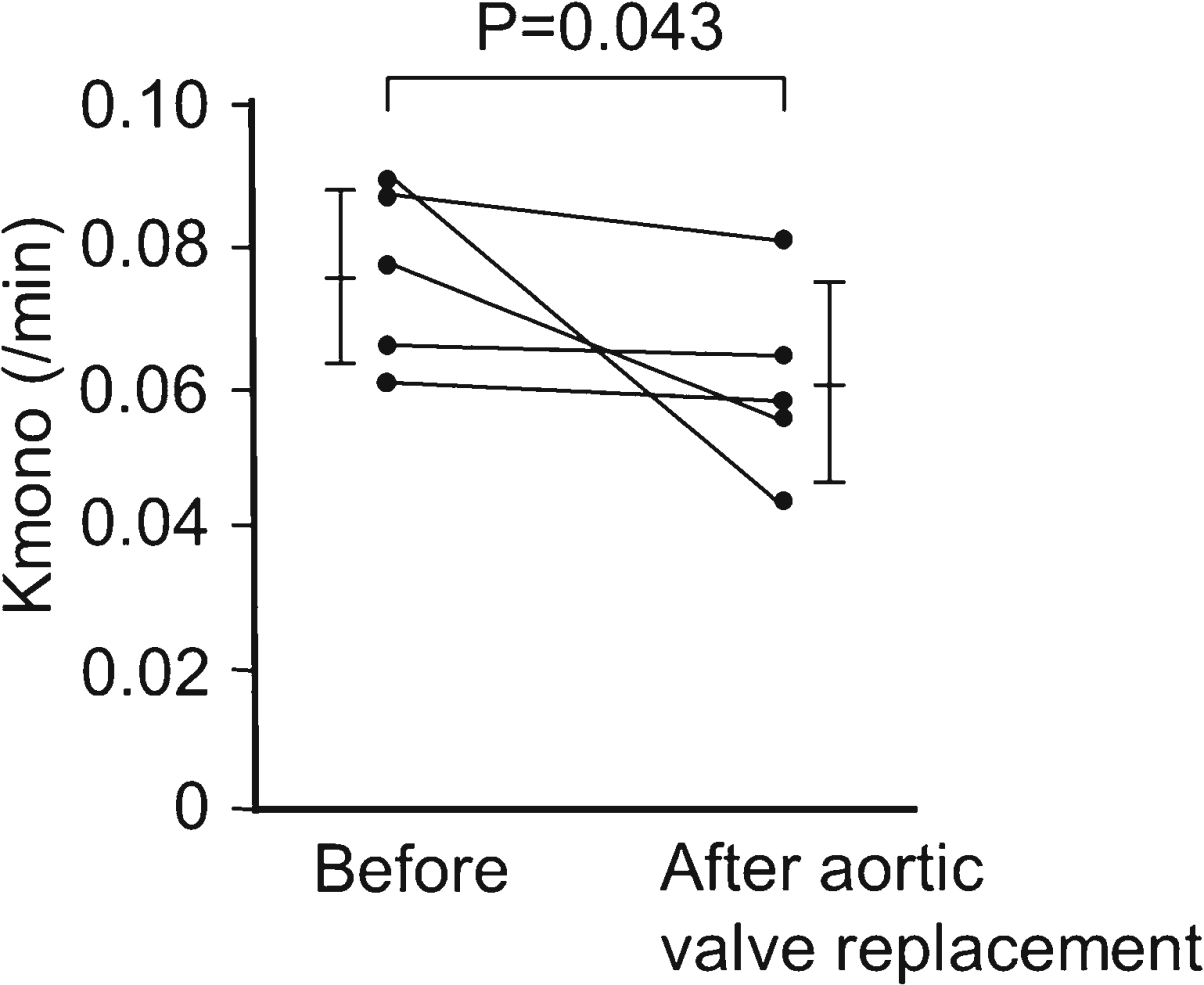
Changes in Kmono before and 1 month after aortic valve replacement in patients with aortic stenosis (n = 5) [37••]

Myocardial oxidative metabolism measured ^11^C-acetate PET is significantly increased by about 40 % in patients with moderate to severe aortic stenosis and preserved systolic volume index compared with healthy controls [36, 37••]. Estimated systolic left ventricular pressure measured by echocardiography was independently associated with the increased myocardial oxidative metabolism (Fig. 2). Importantly, aortic valve replacement ameliorates left ventricular hypertrophy as well as increased oxidative metabolism in patients with aortic stenosis (Fig. 3), indicating that Kmono may be interpreted as a new indicator of myocardial wall stress. Selection of aortic stenosis patients for surgery is generally determined by their symptoms. However, there are many clinical settings such as age and physical disability in which the optimal timing of surgery can be hardly determined. Thus, Kmono estimation may help clinicians to determine the optimal timing for aortic valve replacement for these patients, which, however, needs to be assessed by a well-designed clinical study in the future.

### Effects of Medical Therapy on Work Metabolic Index in Left Ventricular Dysfunction

The concept of work metabolic index estimated by the combination of ^11^C-acetate PET and hemodynamics has been raised by Beanlands et al. [11]. The work metabolic index was determined as follow: Work metabolic index = (systolic volume index * systolic blood pressure *heart rate)/ kmono. This equation is a modification of the equation for left ventricular mechanical efficiency described by Bing et al. [38] and well validated with coronary sinus flow and oxygen consumption measurements by using pulmonary artery and coronary sinus catheter.

**Fig. 4:**
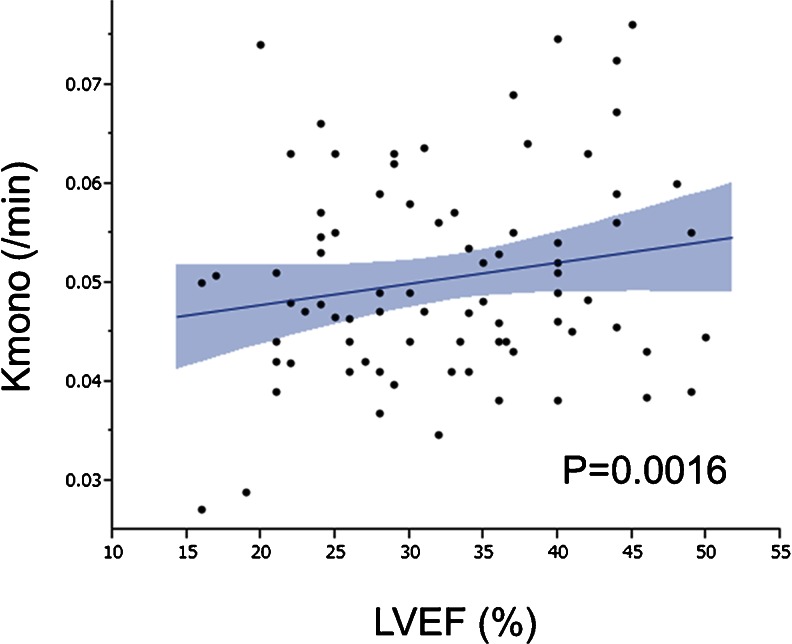
Correlation between left ventricular ejection fraction and global myocardial oxidative metabolism in patients with left ventricular dysfunction [35]

**Fig. 5:**
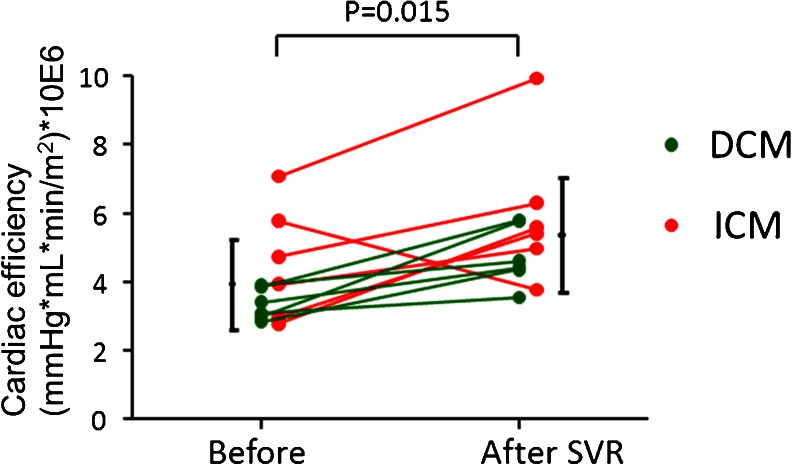
Work metabolic index in patients with dilated cardiomyopathy (DCM) and ICM before and after surgery. The work metabolic index increased in all patients with DCM and in 5 of 6 patients with ICM [43••]

In patients with systolic dysfunction, there is a modest inverse relationship between Kmono and LVEF (Fig. 4). Especially, severe systolic dysfunction is related to low work metabolic index. This work metabolic index seems to be expected to closely relate with the progression of LV remodeling leading to cardiac death. Beanlands et al. demonstrated the excellent study looking at the effects of beta-blockade on the work metabolic index in a double control study. Three months after therapy, Kmono is significantly decreased by 24 % in metoprolol group compared to placebo group (5 % reduction) without change in blood pressure and systolic volume index, results in significant increase in work metabolic index (39 % increase) by metoprolol. Similarly, exercise training improves work metabolic index in patients with dilated cardiomyopathy [39]. They found that exercise training prescribed as 70 % of peak VO2 for 45 mins twice a weak significantly increases work metabolic index by 24.3 %, which is significantly higher than that of control group (4.3 %). In patients who have severe systolic and wide QRS complex, cardiac resynchronized therapy is effective treatment to increase work metabolic index [40, 41], which is caused by homogeneity of metabolism in left ventricle by cardiac resynchronized therapy. These results suggest that intensive medical therapy for treatment of heart failure improves cardiac function in part via improvement of work metabolic index. In patients with refractory heart failure under the optical medical therapy, surgical ventricular reconstruction and mitral complex reconstruction is another therapy [42] although the surgery is not proven to improve outcome. In a pilot study demonstrated by Sugiki et al., the surgery improves the work metabolic index in all six patients with non-ischemic dilated cardiomyopathy and five of six patients with ischemic cardiomyopathy, indicating that the new integrated procedure can be applied for dilated cardiomyopathy [43••] (Fig. 5). However, the optimal timing for our procedure and responders warranted to be investigated in a larger population.

### Mitral Regurgitation

Chow et al. demonstrates the effects of mitral valve surgery on “forward work metabolic index in patients with severe mitral regurgitation [44]. The authors calculate a regurgitation work index (regurgitation volume * peak left atrial pressure / body surface area). In patients with significant mitral regurgitation (regurgitation volume = 34 +/− 19 mL), the index is 0.705 +/− 0.176*10^6^. This is 12 % of total work metabolic index (forward plus regurgitation). After mitral valve surgery, this regurgitation work index becomes almost zero as expect. Accordingly, forward work metabolic index significantly increases from 4.99 +/− 1.32 *10^6^ to 6.59 +/− 2.45 * 10^6^. This study clarifies the effects of volume overload of the left ventricle on cardiac metabolism and efficiency.

#### Oxidative Metabolism in Right Ventricle

C-11 acetate PET is useful to estimate right ventricular oxidative metabolism although catheter is limited to estimate the metabolism due to the complex morphology of right ventricle [45]. In this study, C-11 clearance of right ventricular free wall rate is correlated with the product of systolic pulmonary artery pressure and heart rate for all patients (r = 0.65, p = 0.002), but the relation was stronger if two patients with overt RV dysfunction were excluded (r = 0.83, p = 0.001), suggesting that C-11 acetate PET may allow the assessment of RV metabolism in patients with right ventricular dysfunction. This could be applied in adult congenital heart diseases and severe heart failure under left ventricular-assist device, and the effects of therapeutic interventions on right ventricular dysfunction. The assessment of right ventricular oxidative metabolism is also variable to understand the wall stress of right ventricular wall in patients with idiopathic pulmonary arterial hypertension. Wong et al. reported about a series of 26 patients, who underwent C-11 acetate and PET [46•]. They observed a positive relationship between right ventricular Kmono and rate-pressure product of pulmonary artery in patients with idiopathic pulmonary arterial hypertension. This finding is important to understand the effects of pressure overload on metabolism in right ventricle.

## Conclusions

C-11 labeled compounds have unique character to radiolabeled natural compound, physiological and biochemical processes can be assessed in vivo. If particular, C-11 acetate are both rapidly taken up in the myocardium and incorporated in the metabolic process to excrete from the myocardium as C-11 carbon dioxide. Therefore, the turnover rate of these tracers can reflect oxidative metabolism. This technique provides precise insights into pathophysiology in various conditions of myocardial ischemia and heart failure. Oxidative metabolism is a fundamental major energy source in the myocardium. Thus, oxidative metabolic analysis should play an important role for assessing myocardial viability and treatment effects in the patients with coronary artery disease. Furthermore, C-11 acetate PET has recently been used for metabolic efficiency in heart failure. Clinical impacts of such studies are warranted in the future.
